# Photo-Cross-Linked Porous Hybrid Networks Based on Insoluble Collagen and Poly(trimethylene carbonate)

**DOI:** 10.1021/acs.biomac.5c00971

**Published:** 2025-07-29

**Authors:** Bas van Bochove, Lieke H.A. van Dommelen, Anne-Constance Macarez, Marc Ankoné, Elly M.M. Versteeg, Toin H. van Kuppevelt, Willeke F. Daamen, André A. Poot, Dirk W. Grijpma

**Affiliations:** † Advanced Organ bioengineering and Therapeutics, Department of Bioengineering Technologies, Techmed Centre, 3230 University of Twente, Enschede 7522 NB, The Netherlands; ‡ Department of Medical BioSciences, Research Institute for Medical Innovation, 6034 Radboud university medical center, Nijmegen 6525 GA, The Netherlands

## Abstract

Porous hybrid networks were formed from methacrylated collagen (ICol-MA) fibrils and methacrylated PTMC (PTMC-tMA). Dispersing ICol-MA and dissolving PTMC-tMA in DMSO acidified with HCl (DMSO/HCl), followed by mixing, casting, freezing, photo-cross-linking, and solvent extraction, resulted in networks with high porosities and gel contents. ATR-FTIR showed that hybrid networks had compositions similar to the mixture compositions prior to photo-cross-linking. Mechanical testing of porous hybrid networks, in particular the ICol-MA:PTMC-tMA 17:83 wt % network, showed improved mechanical properties compared to non-cross-linked ICol and ICol-MA networks prepared in DMSO/HCl. Additionally, cross-linking alone already improves the properties of porous collagen structures. Interestingly, photo-cross-linking ICol-MA in acetic acid solution resulted in the best mechanical properties, suggesting that the solvent affects collagen fibril structure and thus network mechanical properties. Indeed, acetic acid does not alter the collagen banding structure, whereas DMSO/HCl does. Thus, further investigation into the effect of the solvent on the network properties is needed.

## Graphical abstract

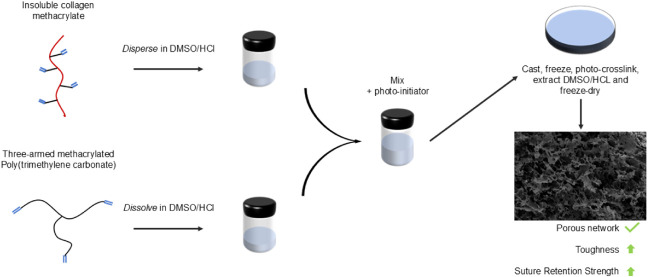

## 1 Introduction

Insoluble type I collagen is a natural polymer that promotes cell adhesion and proliferation^1,2^ and is hemostatic^3^ and biodegradable,^4^ making it particularly relevant for medical applications. In addition, it is naturally present in the human body in large quantities. However, porous scaffolds prepared from insoluble collagen are known for their poor mechanical properties,^5^ in particular if it has been pulverized to allow purification. While collagen presents a good alternative to other natural polymers, studies in larger animal models and thus testing for clinical applications have been limited due to the aforementioned subpar mechanical properties.

Cross-linking provides an excellent method to prepare networks with improved mechanical properties and increased stability.^5^ Several methods to cross-link collagen have been reported. One method involves chemical cross-linking using 1-ethyl-3-(3-dimethylaminopropyl)­carbodiimide (EDC) and *N*-hydroxysuccinimide (NHS).^4^ In addition, photo-cross-linking by chemically modifying soluble collagen with a (meth)­acrylate has been reported.^6–9^ While this somewhat improved the mechanical properties of collagen structures, the obtained collagen networks remained relatively fragile and brittle.

A novel strategy to overcome unsatisfactory mechanical properties of networks prepared from hydrophilic natural polymers is the preparation of hybrid networks from these natural polymers with hydrophobic synthetic polymers.^10–12^ By methacrylate functionalization, the polymers can be modified to allow for photo-cross-linking. The functionalized compounds are dissolved or dispersed in a common solvent/dispersion agent. They are then cast and photo-cross-linked. Once the networks have formed, the solvent and unreacted compounds are extracted, and the materials are dried.

Previous studies have shown that combining a natural hydrophilic compound with hydrophobic synthetic poly­(trimethylene carbonate) (PTMC) by photo-cross-linking results in hybrid networks with improved mechanical and biological properties, such as elasticity, flexibility, and compatibility with different types of cells,^10,12^ as compared to their respective individual networks. PTMC is a biocompatible and biodegradable polymer. Photo-cross-linked networks prepared from PTMC have excellent mechanical properties, which are dependent on the macromer molecular weight, and they degrade by enzymatic surface erosion. These properties have enabled the use of PTMC networks in regenerative medicine studies.^10,13,14^ We hypothesize that photo-cross-linking methacrylated insoluble collagen fibrils and PTMC into porous hybrid networks is a viable strategy to improve the poor mechanical properties of porous collagen structures.

## 2 Materials and Methods

### 2.1 Materials

Trimethylene carbonate (TMC) was supplied by Huizhou Foryou Medical Devices (China). Tin­(II) 2-ethylhexanoate (Sn­(Oct)_2_), trimethylolpropane (TMP, 2-ethyl-2-(hydroxymethyl)­propane-1,3-diol), hydroquinone (benzene-1,4-diol), methacrylic anhydride (2-methylprop-2-enoic anhydride), triethylamine (*N*,*N*-diethylethanamine), glycidyl methacrylate (oxiran-2-ylmethyl 2-methylprop-2-enoate), 2-hydroxy-4’-(2-hydroxyethoxy)-2-methylpropiophenone (Irgacure-2959), hydroxylamine hydrochloride, sodium hydroxide (NaOH), Fe­(III)-perchloride, acetohydroxamic acid, acetic acid, d-chloroform, and hydrochloric acid (HCl) were purchased from Sigma-Aldrich (USA). Dichloromethane (DCM), dimethyl sulfoxide (DMSO), and sodium chloride (NaCl) were obtained from VWR Chemicals (Netherlands). Phosphate-buffered saline (PBS), 2-morpholinoethanesulfonic acid (MES), ethanol, EDC, and disodium hydrogen phosphate (Na_2_HPO_4_) were purchased from Merck (Germany). NHS was obtained from Fluka Chemie AG (Switzerland). Insoluble collagen type I (ICol) fibrils were isolated from bovine tendon.^4,15^

### 2.2 Methacrylate Functionalization of Insoluble Collagen

ICol (0.5% (w/v) in 0.25 M acetic acid) was functionalized by reaction with glycidyl methacrylate (10 mL/g) for 1 day under continuous stirring at room temperature (RT) in the dark (Scheme 1). Dialysis was carried out to extract the acetic acid and unreacted glycidyl methacrylate to obtain methacrylated insoluble collagen (ICol-MA). The solution was transferred to semipermeable membranes (Spectra/Por 4 dialysis membrane, molecular weight cutoff of 12–14 kDa), previously immersed in 0.25 M acetic acid, which were introduced into a bath of 0.25 M acetic acid for 24 h and then in water for 2 days (with 2–3 changes of the solutions per day). Finally, ICol-MA was freeze-dried.

**Scheme 1:**
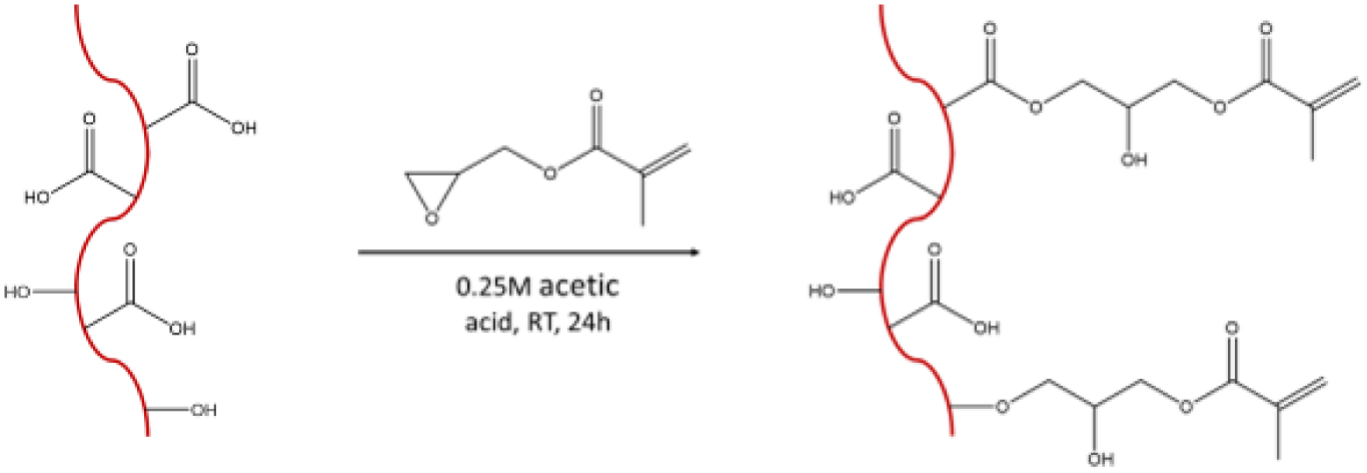
Functionalization of Insoluble Collagen (ICol) with Glycidyl Methacrylate in 0.25 M Acetic Acid to Obtain ICol-MA

The degree of functionalization of the ICol-MA was determined as reported by Yue et al.^16^ 40 mg of methacrylated collagen was dissolved in 800 μL of PBS, with the subsequent addition of 800 μL of a 1:1 mixture of hydroxylamine hydrochloride (0.5 mol/L) and NaOH (1.0 mol/L). The solution was vortexed for 30 s and left at RT for 10 min. 2200 μL of HCl (0.5 mol/L) and 200 μL of an Fe­(III)-perchloride solution (0.5 mol/L in 0.5 mol/L HCl) were added to the initial solution and vortexed for 30 s. The mixture was centrifuged for 1 min at 12,880 g before measuring the absorbance at 500 nm by UV–vis (Cary 300 UV–vis Spectrophotometer, Agilent Technologies) using a calibration curve of a series of acetohydroxamic acid solutions (ranging from 0 to 1.25 mmol/L) prepared by mixing 5 mmol/L acetohydroxamic acid with demineralized water and Fe­(III)-perchloride solution (0.5 mol/L in 0.5 mol/L HCl).

The degree of functionalization (DF) of ICol-MA was calculated by considering the number of hydroxyl and carboxyl groups present in insoluble type I collagen^4^ using the following equation:

1 $$DF=\frac{\mathrm{mmol}\;\mathrm{methacrylate}}{\mathrm{mmol}\;\mathrm{COOH}\;\mathrm{and}\;\mathrm{OH}\;\mathrm{groups}}\times 100\%$$

### 2.3 Synthesis of Methacrylated Poly­(trimethylene carbonate)

A three-armed PTMC oligomer was synthesized by ring-opening polymerization of TMC to obtain an intended molecular weight of 20 kg/mol. 50 g of TMC was placed in a three-neck flask, mixed with 0.33 g of TMP (as initiator), and heated to 130 °C under argon. After melting and mixing, Sn­(Oct)_2_ (5 × 10^–5^ mol/mol monomer) was added as a catalyst, and polymerization was carried out for 3 days. The monomer conversion and oligomer molecular weight were determined by ^1^H NMR spectroscopy in d-chloroform as described.^17^

Subsequently, the oligomer was dissolved in dried and distilled DCM (4 mL/g of monomer). Functionalization was performed by adding 2.78 mL of methacrylic anhydride and 2.61 mL of triethylamine to the solution. Next, 0.05 g of hydroquinone was added to inhibit premature cross-linking. The reaction was carried out for 7 days in the dark at RT. The obtained methacrylated PTMC oligomer (macromer, PTMC-tMA) was precipitated in cold ethanol and dried under a vacuum. The degree of functionalization was determined by ^1^H NMR spectroscopy.^17^ Scheme 2 provides an overview of the polymerization and functionalization reaction.

**Scheme 2:**
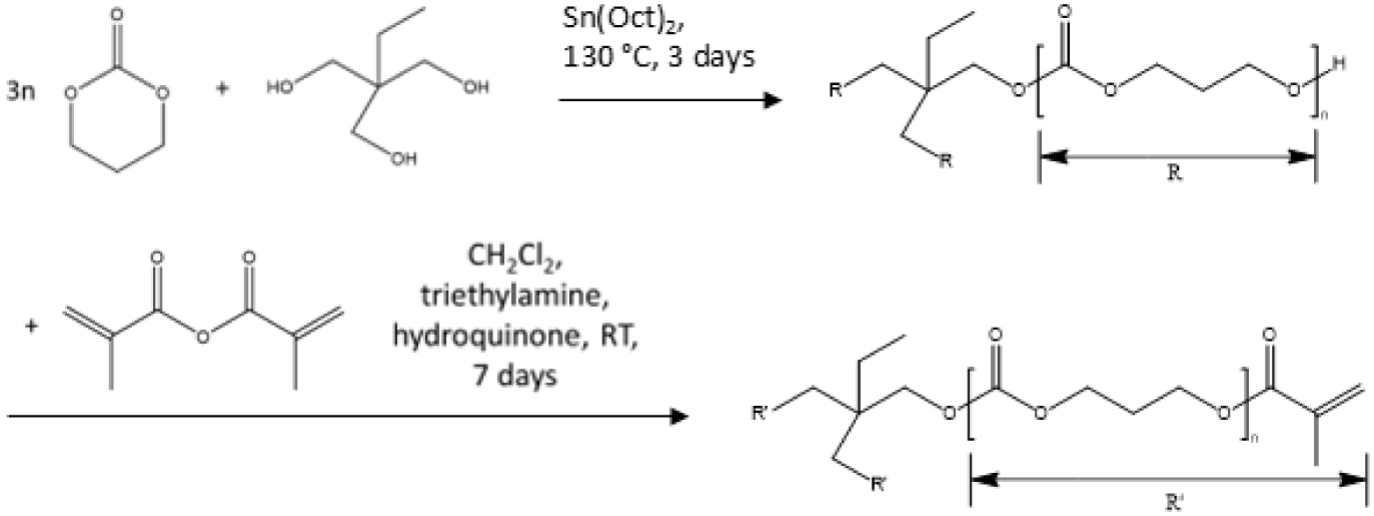
Ring-Opening Polymerization of Three-Armed PTMC Using TMP as Initiator, Followed by Functionalisation with Methacrylic Anhydride to Obtain PTMC-tMA

### 2.4 Preparation of Porous Collagen Structures

#### 2.4.1 Preparation of a Non-Cross-Linked Collagen Structure

A non-cross-linked collagen structure was prepared by swelling ICol in 0.25 M acetic acid (0.8% w/v) at 4 °C for 16 h. Subsequently, the ICol was homogenized using a Potter–Elvehjem homogenizer (Lauwers Glass and Ceramic Technologies, Hapert, The Netherlands). The ICol dispersion was then poured into a polystyrene Petri dish, frozen at −20 °C, and freeze-dried.

#### 2.4.2 Preparation of EDC/NHS-Cross-Linked ICol Networks

Cross-linked ICol networks were prepared by chemical cross-linking using EDC and NHS.^4^ The reaction is shown in Scheme 3. Briefly, non-cross-linked collagen structures were incubated for 30 min at RT in 50 mM MES containing 40% (v/v) ethanol (MES buffer, pH 5.0) and subsequently cross-linked for 4 h in 50 mM MES buffer containing 33 mM EDC and 6 mM NHS. Then, the networks were washed twice in 0.1 M Na_2_HPO_4_, twice in 1 M NaCl, and twice in 2 M NaCl, followed by six washings with distilled water and freeze-drying.

**Scheme 3:**
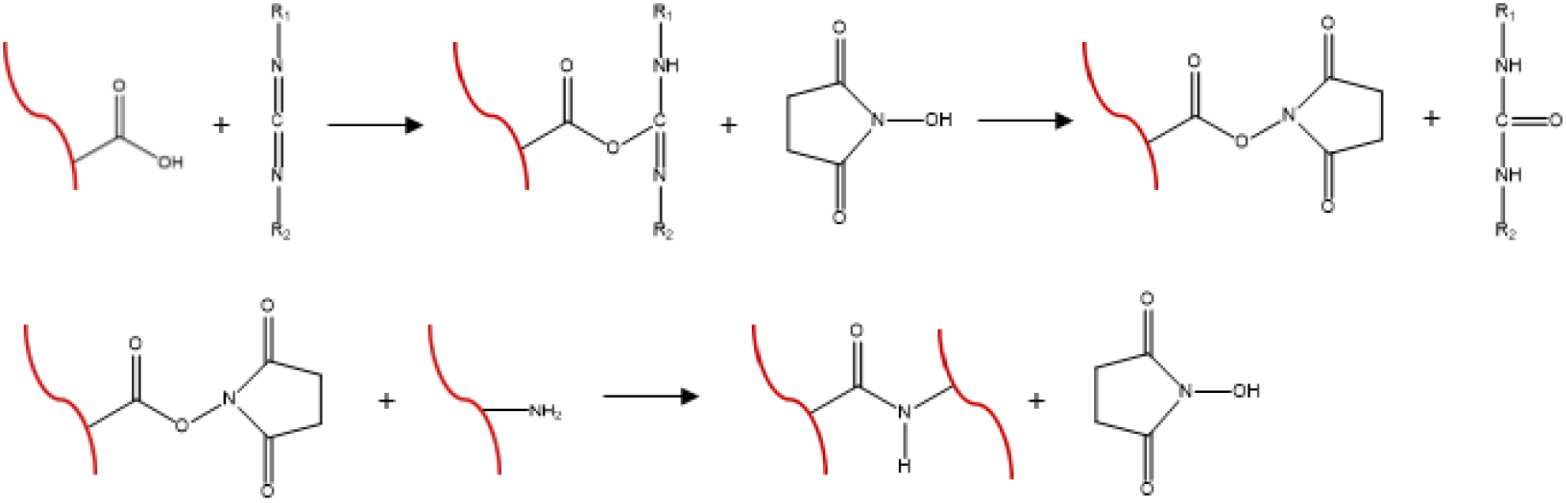
Chemical Crosslinking of Insoluble Collagen with EDC and NHS

#### 2.4.3 Preparation of Photo-Cross-Linked ICol Networks

ICol-MA was cut and swollen for 1 day at a concentration of 3% (w/v) in a solution of DMSO and HCl (DMSO/HCl, with a theoretical pH of 2) while stirring and protected from light at RT. The obtained collagen dispersion was homogenized using a Potter–Elvehjem homogenizer, and Irgacure-2959 (6 wt % relative to the ICol-MA) was added as a photoinitiator and mixed for 1 h. The mixture was cast and placed in a freezer at −25 °C overnight before being photo-cross-linked in the frozen state by UV irradiation for 1 h in a UV chamber (365 nm) on a −15 °C plate.

After photo-cross-linking, the DMSO/HCl and unreacted collagen were extracted. The obtained networks were first placed in DMSO/HCl. Then, demineralized water was gradually added at approximately 1 mL/min for 24 h, slowly replacing the DMSO/HCl. Subsequently, the water was removed by freezing in a freezer at −25 °C overnight and freeze-drying.

Alternatively, ICol-MA was cut and swollen at a concentration of 0.9% (w/v) in 0.25 M acetic acid. Photo-cross-linked networks were subsequently prepared as described before. After cross-linking, acetic acid and unreacted collagen were extracted by solvent exchange to water and freeze-drying.

### 2.5 Preparation of Porous PTMC and Hybrid Networks

#### 2.5.1 Preparation of PTMC Networks

The PTMC-tMA was dissolved in a solution of DMSO/HCl at a concentration of 15% (w/v) by stirring for 24 h at 70 °C. One wt% Irgacure-2959 relative to PTMC-tMA was added and mixed for 1 h. The solution was cast and frozen at −25 °C overnight and subsequently cross-linked for 1 h in a UV chamber (365 nm) on a −15 °C plate. The networks were extracted as described for collagen networks and dried by freezing at −25 °C overnight, followed by freeze-drying.

#### 2.5.2 Preparation of ICol-MA/PTMC-tMA Hybrid Networks

The ICol-MA dispersions and PTMC-tMA solutions were prepared separately in DMSO/HCl by mixing for 24 h at concentrations of 6% (w/v) for the collagen and 10% (w/v) and 30% (w/v) for the PTMC. Two hybrid mixtures were prepared by mixing equal volumes of the collagen dispersion and a PTMC solution. The final concentrations were 3% (w/v) and 5% (w/v) of ICol-MA and PTMC-tMA, respectively, for the first mixture and 3% (w/v) and 15% (w/v) of ICol-MA and PTMC-tMA, respectively, for the second mixture. After homogenization of the mixtures, Irgacure-2959 was added (1 wt % relative to the PTMC-tMA and 6 wt % relative to ICol-MA) and mixed for 1 h. Subsequent casting, freezing, cross-linking, extraction, and freeze-drying were performed as described in Section 2.4.3.

### 2.6 Network Characterization

#### 2.6.1 Gel Content and Water Content

The gel content *G* was determined by taking the mass values of the polymers before solvent extraction (*m*_total_) and after drying (*m*_d_) (eqs 2 and 3):

2 $$G=\frac{m_{d}}{m_{1}}\times 100\%$$

with

3 $$m_{1}=m_{\mathrm{total}}\times \left(\frac{m_{\mathrm{initial}}}{m_{\mathrm{initial}}+m_{\mathrm{solvent}}}\right)$$

where *m*_initial_is the polymer mass that is initially dispersed or dissolved in DMSO/HCl and *m*_solvent_ is the mass of DMSO/HCl.

To calculate the water content, each extracted material was weighed dry (*m*_d_ *)* and swollen (*m*_s_). The swollen mass was taken after the networks had been placed in demineralized water for 48 h. The water content was then determined by the following equation:

4 $$\mathrm{Water}\;\mathrm{content}=\frac{m_{s}-m_{d}}{m_{d}}\times 100\%$$

#### 2.6.2 Porosity

The porosity of the obtained networks was obtained by weighing three samples of each network. The volume was determined by measuring the length, width, and thickness of each sample (approximately 5 × 5 mm, with thickness varying between the types of networks). The porosity was calculated using the following equation:

5 $$\mathrm{Porosity}=\left(1-\frac{m}{V\times \rho }\right)\times 100\%$$

where *m* is the mass of the sample in g, *V* is the volume in cm^3^, and ρ is the density of the polymers in g/cm^3^ (1.3 g/cm^3^ for both collagen^18^ and PTMC^13^).

#### 2.6.3 Network and Fibril Morphology

The morphologies of the cross sections of the networks were investigated by scanning electron microscopy (SEM, JEOL JSM-IT100) at 5.0 kV. The extracted and dried networks were immersed in liquid nitrogen and subsequently cut in one smooth movement to prevent compression and alter the morphology. Samples were gold-sputtered using a Cressington Sputter Coater 108 auto with a pure gold target at 10 mA for 60 s.

The morphologies of the ICol-MA fibrils were investigated by SEM (Zeiss Sigma 300) at an accelerating voltage of 3.0 kV. Untreated ICol-MA and ICol-MA immersed in DMSO or DMSO/HCl and washed with water were sputter-coated for 60 s with gold using an Edwards Scancoat Six sputter coater.

#### 2.6.4 Thermal Properties

The thermal properties of the obtained (hybrid) networks were studied by thermal gravimetric analysis (TGA) and differential scanning calorimetry (DSC) using N_2_ as an inert gas.

By TGA, the decomposition temperature was determined. Samples between 2 and 3 mg were heated from 30 to 600 °C at a rate of 10 °C/min.

DSC was used to determine the glass transition temperature (*T*_g_). Samples between 3 and 5 mg were first cooled to −60 °C and subsequently heated at 10 °C/min up to 150 °C. Next, the samples were cooled to −60 °C and again heated at 10 °C/min up to 150 °C. *T*_g_ values were taken from the second heating scan.

#### 2.6.5 Hybrid Network Composition

The hybrid network composition was determined by attenuated total reflectance Fourier transform infrared spectroscopy (ATR-FTIR, Spectrum Two, PerkinElmer). The resolution was 4 cm^–1^, and 8 scans were made between 4000 and 400 cm^–1^. The peaks at ∼1540 cm^–1^ correspond to collagen amide II groups,^19^ and those at ∼1740 cm^–1^ correspond to the PTMC C=O groups.^10^ From the absorption spectra, the heights of these characteristic peaks were determined to make a semiquantification of the ratio of each polymer in the hybrid networks.

#### 2.6.6 Mechanical Properties

Tensile stress–strain measurements were performed using a TA Instruments DMA 850 instrument with samples of each network in the dry and hydrated state. For the hydrated state, networks were placed in demineralized water for 48 h before being subjected to stress. Samples were strips with an approximate thickness and width of 0.8 and 2.5 mm, respectively, and an approximate length of 2.05 cm. The elastic modulus, maximum stress (σ_max_), and elongation at break (ε_break_) were determined at a test speed of 0.5 mm/min. The modulus was determined by calculating the slope of the tangent of the steepest part of the stress–strain curve. The toughness (*W*) was calculated as the area under the stress–strain curve.

The suture retention strength (SRS) of the networks was determined in both the dry and hydrated state. Samples with an approximate thickness and width of 0.8 and 2.5 mm, respectively, and an approximate length of 1.5 cm were used. Two mm from the top of a sample, a hole was made using a syringe needle (25G), followed by the insertion of a stainless-steel wire (0.1 mm diameter, Monacor, Germany) through the hole. The ends of the wire were clamped in the upper grip of the DMA 850, while the other side of the sample was clamped in the lower grip. The DMA was operating in the tensile mode with a crosshead speed of 0.5 mm/min. The SRS was obtained by normalizing the values for the sample thickness and is given in N/mm.

## 3 Results and Discussion

### 3.1 Methacrylate Functionalization of Collagen

The methacrylate functionalization of ICol was done in an acidic medium, which allows for the reaction of glycidyl methacrylate with hydroxyl and carboxylic acid groups of the ICol.^20^ The DF of IColMA was determined by an Fe­(III)-hydroxamic acid assay to be 24%. The calibration curve of the assay can be found in Figure S1. Due to the large number of hydroxyl and carboxylic acid groups in a collagen molecule,^4^ this DF is more than sufficient for adequate photo-cross-linking and gel formation.

### 3.2 Synthesis of Methacrylated Poly­(trimethylene carbonate)

The PTMC oligomer was made by ring-opening polymerization of TMC, with TMP as the initiator. ^1^H NMR spectra enabled us to identify the molar mass and DF, as described.^13,17^

We aimed for an *M*_n_ of 20 kg/mol as these oligomers are still easy to dissolve; the solutions are not too viscous and therefore still relatively easy to cross-link. Using the integral values of the peak of the −CH_2_– methylene groups of the PTMC at δ 4.24 ppm and the −CH_3_– groups of the initiator at δ 0.92 ppm, we calculated the molar mass (*M*_n_) of the oligomer to be 21 kg/mol. By comparing the integral values of the PTMC peak at δ 4.24 ppm and the TMC peak at δ 4.47 ppm, we determined the monomer conversion to be 99%.

Following the subsequent functionalization, the DF of PTMC-tMA was determined using the integral values of the peak of the −CH_3_– groups of the initiator at δ 0.92 ppm and the methacrylate groups at δ 6.11 and 5.57 ppm. The DF of PTMC-tMA was 96%.

### 3.3 Network Preparation

The challenge in preparing hybrid networks from a hydrophilic natural polymer and a hydrophobic synthetic polymer is finding a common solvent. For the work presented here, this was a particular challenge as the collagen used is insoluble. Aqueous acetic acid has been used to disperse ICol for the preparation of non-cross-linked samples,^4^ and in the present study, it has been used for methacrylation. However, PTMC does not dissolve or disperse in dilute acetic acid. In previous work on hybrid networks prepared from PTMC and hyaluronic acid, it was found that DMSO/HCl could dissolve both polymers.^10,12^ We found that ICol-MA could be dispersed in this medium up to at least 6 w/v%. This allowed us to prepare hybrid mixtures containing dispersed ICol-MA and dissolved PTMC-tMA. After homogenization and the addition of a photoinitiator, the mixtures were frozen at −25 °C. This crystallizes DMSO/HCl, resulting in polymer-poor phases surrounded by polymer-rich phases, aiding in successful cross-linking at −15 °C. After cross-linking, the crystallized medium can be extracted by water, which can be removed by subsequent freeze-drying. This results in porous networks.

### 3.4 Network characterization

#### 3.4.1 Hybrid Network Composition

The ratio of polymers in the hybrid networks after extraction and drying was examined by ATR-FTIR. Using this semiquantitative method, we previously showed that the composition of hybrid networks may deviate slightly from the intended composition.^10,21^ The gel contents of the photo-cross-linked networks were determined by measuring the mass of the networks directly after cross-linking and after freeze-drying using eqs 2 and 3. All gel contents were above 78%, indicating effective cross-linking. However, as the gel contents of the hybrid networks prepared were lower than 100% (see Table 1), the obtained compositions may indeed be different from the intended ones as it could be possible that one of the two polymers has cross-linked to a lesser extent.

**Table 1:** Network Characteristics

Network	Gel Content (%)	Composition (ICol:PTMC (wt%))	*T*_g_ (°C)
ICol-MA	99.5	100:0	230^a^
ICol-MA:PTMC-tMA 38:62 wt %	87.8	35:65	–14
ICol-MA:PTMC-tMA 17:83 wt %	78.4	17:83	–14
PTMC-tMA	89.4	0:100	–14

a *T*_g_ for type I collagen obtained from the literature.^22^

Figure 1 shows the obtained spectra. The peak at ∼1540 cm^–1^ corresponds to collagen amide II,^19^ and the peak at ∼1740 cm^–1^ corresponds to PTMC C=O groups.^10^ By comparing the height of these characteristic peaks, the network composition was semiquantified. The dispersion containing 3% w/v ICol-MA and 5% w/v PTMC-tMA should lead to networks with a composition of 17:83 wt % ICol:PTMC. This network was found to have exactly that composition. The dispersion with 3% w/v ICol-MA and 15% w/v PTMC-tMA was expected to result in networks with a composition of 38:62 wt % ICol:PTMC. The composition of this network deviated slightly with an obtained ratio of 35:65 wt %.

**Figure 1:**
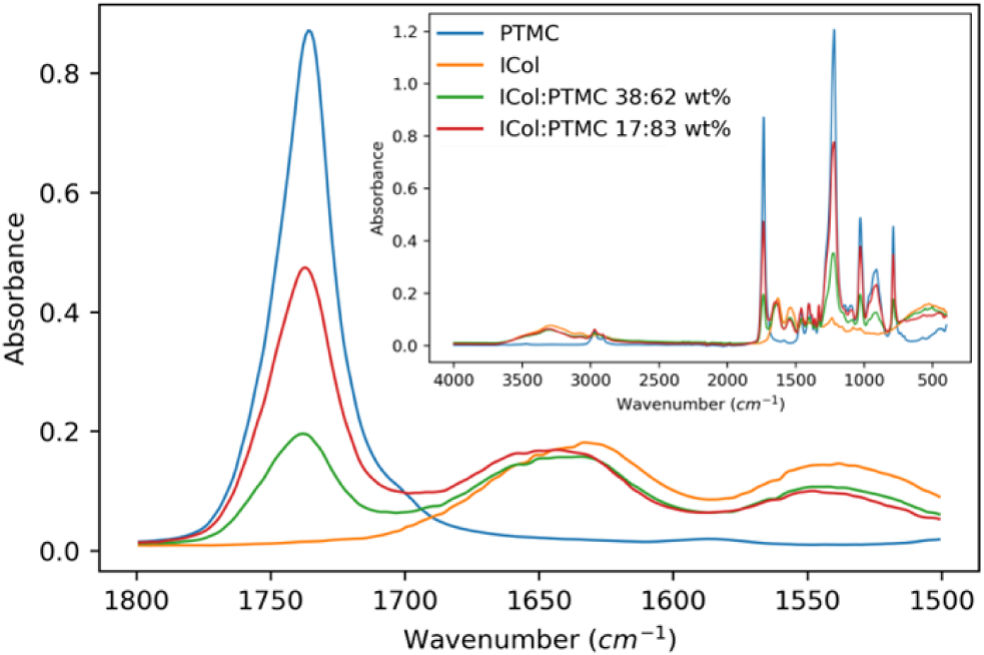
ATR-FTIR spectra of the (hybrid) networks between 1500 and 1800 cm^–1^ showing the characteristic PTMC C=O peak at 1740 cm^–1^ and the characteristic collagen amide II peak at 1540 cm^–1^. The inset shows the full spectra.

#### 3.4.2 Thermal Properties

The thermal properties of the hybrid networks were determined by TGA and DSC. TGA was used to determine the decomposition temperatures and set the upper limit of the DSC measurements. The thermograms are shown in Figure S2. It was clear that apart from water evaporation, decomposition of collagen started at approximately 200 °C. To prevent decomposition in the DSC, the maximum temperature was set at 150 °C. The results of the DSC measurements are shown in Table 1. *T*_g_ of collagen was not observed as the reported value of 230 °C^22^ was outside the measurement range. The hybrid networks showed the same *T*_g_ as the PTMC network, indicating that this is *T*_g_ of the PTMC and thus that the hybrid networks were phase-separated. The phase-separation could not be seen with the naked eye nor with scanning electron microscopy, indicating that homogeneous but phase-separated networks were obtained.

#### 3.4.3 Physical Properties

Due to cross-linking at low temperatures, DMSO/HCl had crystallized. As a result, the networks obtained after extraction and freeze-drying were porous. All samples containing collagen were highly porous, with minimal porosities of 83.2 ± 1.3%. Table 2 gives an overview of all porosities. The PTMC network had a lower porosity of 65.5 ± 4.0%, likely due to network shrinkage during freeze-drying. The porous nature of the photo-cross-linked networks and the non-cross-linked ICol structure was confirmed by SEM; see Figure 2. Only the PTMC network shows a pore morphology that is characterized by long, thin pores. This is similar to the pore morphology of the P­(TMC-co-ε-CL), recombinant human-like collagen (rh-collagen), and their hybrid networks we previously studied.^21^ Likely, the long, thin pore architecture is due to the rate and direction of freezing of DMSO/HCl. PTMC, P­(TMC-co-ε-CL), and rh-collagen are soluble polymers, whereas ICol-MA was dispersed. As can be seen in the morphologies of the ICol scaffold and ICol-MA-containing networks, this results in a less organized pore structure in which the clear long, thin pore morphology is not present.

**Figure 2:**
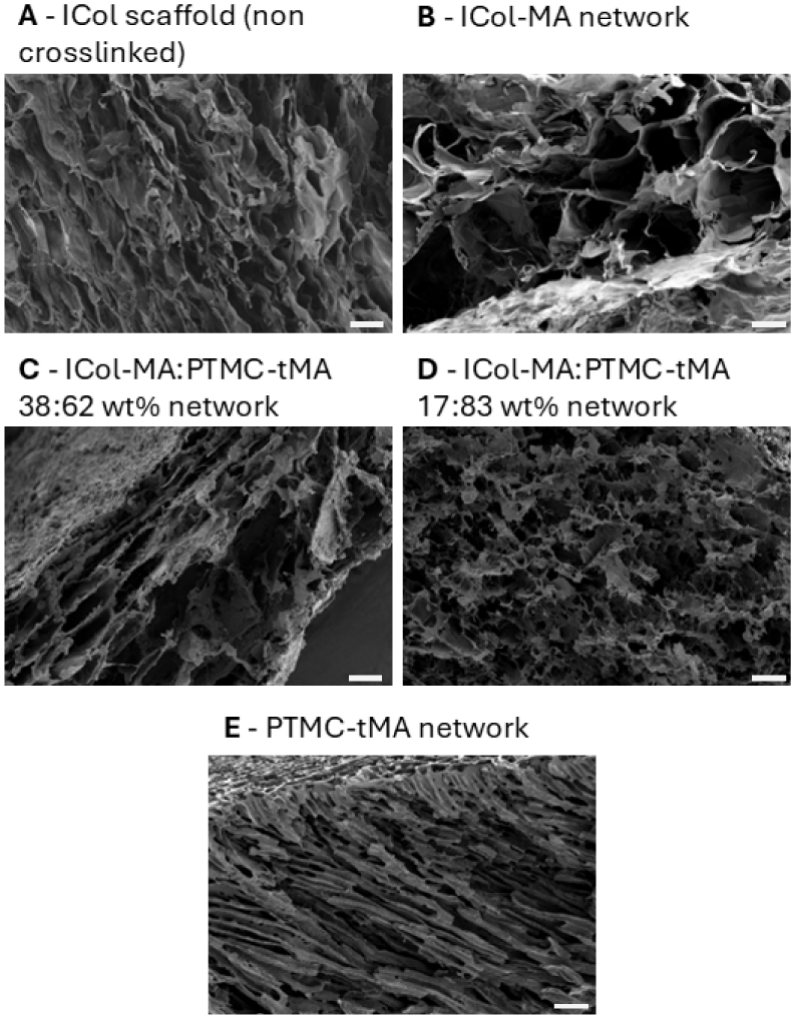
SEM pictures of cross sections of an ICol scaffold and the DMSO/HCl photo-cross-linked (hybrid) networks. The scale bar is 100 μm.

**Table 2:** Porosity and Mechanical Properties of Dry Non-Cross-Linked ICol and Networks Prepared from ICol-MA and/or PTMC-tMA

		**Tensile Properties**				
Material	Porosity (%)	*E*-modulus (MPa)	σ_max_ (N mm^–2^)	ε_break_ (%)	*W* (N mm^–2^)	SRS (N mm^–1^)
ICol (non-cross-linked)	95.6 ± 0.8	5.0 ± 1.4	0.33 ± 0.06	17.1 ± 2.1	2.7 ± 0.4	0.3 ± 0.1
ICol-MA network (in DMSO/HCl)	96.2 ± 0.4	18.0 ± 6.8	0.75 ± 0.13	10.0 ± 3.9	3.3 ± 0.9	0.6 ± 0.1
ICol-MA:PTMC-tMA 38:62 wt % network	89.6 ± 0.7	12.1 ± 4.6	0.45 ± 0.10	10.7 ± 2.9	2.2 ± 0.4	0.6 ± 0.5
ICol-MA:PTMC-tMA 17:83 wt % network	83.2 ± 1.3	4.9 ± 2.6	0.42 ± 0.18	26.6 ± 4.5	4.5 ± 0.7	0.5 ± 0.1
PTMC-tMA network	65.5 ± 4.0	2.2 ± 1.6	>1.14 ± 0.5^a^	>156 ± 8^a^	>55.8 ± 20.8^a^	>1.2 ± 1.0^a^

a Two out of three samples broke. The third sample reached the machine limit and did not break.

The capacity of the networks to contain water was calculated by using eq 4. The ability to contain water is an important feature of networks for tissue engineering, as it provides an indication of the networks to facilitate a hydrated environment important for nutrient supply. The values for the water content are shown in Table 3. The PTMC network had the lowest water content, which is consistent with its hydrophobic nature. The water content was considerably higher as compared to previously reported solid PTMC networks.^10^ This is likely due to the porous nature of the networks used in this study. While samples were blotted dry prior to measuring the swollen mass, the pores in the networks could still contain water, resulting in measuring a higher mass of water than that strictly taken up by the polymer matrix. This was the case for all of the measured samples.

**Table 3:** Water Content and Mechanical Properties of Wet Non-Cross-Linked ICol and Networks Prepared from ICol-Ma and/or PTMC-tMA

		**Tensile Properties**				
Material	Water Content(%)	*E*-modulus (MPa)	σ_max_ (N mm^–2^)	ε_break_ (%)	*W* (N mm^–2^)	SRS (N mm^–1^)
ICol (non-cross-linked)	4036 ± 281	0.05 ± 0.02	0.01 ± 0.003	51.1 ± 11.4	0.2 ± 0.1	0.01 ± 0.006
ICol-MA network (in DMSO/HCl)	2018 ± 341	0.27 ± 0.09	0.06 ± 0.02	62.5 ± 8.3	1.6 ± 0.6	0.2 ± 0.08
ICol-MA:PTMC-tMA 38:62 wt % network	798 ± 48	0.19 ± 0.09	0.07 ± 0.04	64.9 ± 12.0	1.9 ± 1.2	0.1 ± 0.03
ICol-MA:PTMC-tMA 17:83 wt % network	608 ± 87	0.17 ± 0.02	0.06 ± 0.01	76.2 ± 6.2	2.0 ± 0.2	0.3 ± 0.3
PTMC-tMA network	128 ± 6	2.0 ± 0.5	>0.99 ± 0.20^a^	>159 ± 2^a^	>49.1 ± 9.5^a^	>1.6 ± 1.4^b^

a No samples broke.

b Two out of three samples broke. The third sample reached the machine limit and did not break.

The addition of collagen to the networks increased the hydrophilic characteristics and, therefore, the capacity of the structures to hold water. These results were confirmed by an increasing water content with increasing collagen content in the networks. The differences between the two hybrid networks were small. These results are in line with water contents of hybrid networks we previously reported.^10–12^ The hydrophilic nature of collagen is shown by the high water content of the cross-linked ICol-MA network, which was 2–3-fold higher than that of the hybrid networks. It does seem that cross-linking restricts the water content of collagen, as the cross-linked ICol-MA network had a water content approximately half of that of the non-cross-linked ICol structure.

#### 3.4.4 Mechanical Properties

The mechanical properties of the networks were determined by tensile testing and suture retention strength (SRS) measurements in the tensile mode. An overview of the results is shown in Table 2 (dry networks) and Table 3 (wet networks). Note that most of the PTMC networks did not break before the maximum path length during the measurements was reached.

In the dry state, the ICol-MA network had a considerably higher elastic modulus than those of the other networks. The average modulus decreased with an increasing PTMC content. The elongation at break increased with an increasing PTMC content. This suggests that increasing the PTMC content results in a less brittle and more ductile network. An important and often overlooked property is that of the toughness of the networks or the energy required to break the networks. While the differences were relatively small, the trend suggests increasing toughness with increasing PTMC content.

A more relevant state of measurement of mechanical properties for tissue engineering applications is the wet state. Among the photo-cross-linked networks, a trend with higher average toughness was observed for the hybrid networks compared to the ICol-MA network.

Interestingly, the average SRS of the wet ICol-MA network was higher than that of the ICol-MA:PTMC-tMA 38:62 wt % hybrid network. This is likely due to water bridges and hydrogen bonds formed by the water between the collagen bundles, strengthening the networks and increasing the amount of energy absorbed by the networks during deformation. The ICol-MA:PTMC-tMA 17:83 wt % hybrid network showed a limited increase in toughness and SRS compared to the ICol-MA network, indicating that the hybrid networks are interesting for tissue engineering applications that require suturing. The results also highlight the need to further optimize the ratio between ICol-MA and PTMC-tMA in the networks. A higher PTMC content will likely increase the toughness and SRS of the hybrid networks further. Furthermore, our preliminary data (not shown) of degradation experiments indicate that the degradation of networks prepared from ICol-MA is much faster than that of hybrid networks. Hybrid networks are therefore likely to allow more time for tissue ingrowth in tissue engineering applications, such as patches for diaphragmatic hernia closure.

Some limitations that influence the measurement of the mechanical properties must be discussed. All samples in this study were porous. Ideally, solid samples would have been used, but at the low concentrations of these solutions, cross-linking in the liquid state (above the melting temperature of DMSO) did not work, which meant we could not obtain nonporous samples. The difference in material concentrations resulted in different porosities of the networks, further affecting the mechanical properties.

### 3.5 Effect of Photo-Cross-Linking

The results of our experiments show that preparing photo-cross-linked hybrid networks results in networks with improved mechanical properties. Interestingly, comparing the non-cross-linked ICol structure with the cross-linked ICol-MA network indicates that cross-linking itself is beneficial, as the modulus, toughness, and SRS of the ICol-MA network iare considerably higher than those of the non-cross-linked ICol structure (see Tables 2 and 3). Therefore, we compared the properties of different cross-linked collagen networks with a non-cross-linked ICol structure. Thus, in addition to the already prepared ICol-MA network cross-linked in DMSO/HCl, a network was prepared by photo-cross-linking ICol-MA in acetic acid solution (as non-cross-linked ICol structures are also prepared in acetic acid solution), and an EDC/NHS-cross-linked ICol network was prepared as described.^4^

#### 3.5.1 Porosity and Water Uptake

The porosity of the networks is shown in Table 4. All materials had similar high porosity, with a minimum porosity of 95.6 ± 0.8%. The water contents of these collagen materials are shown in Table 5. It appears that cross-linking restricts the water content of collagen, as all cross-linked networks had a water content considerably lower than that of the non-cross-linked ICol structure.

**Table 4:** Porosity and Mechanical Properties of Dry Non-Cross-Linked ICol and Networks Prepared from ICol and ICol-Ma

		**Tensile Properties**				
Material	Porosity (%)	*E*-modulus (MPa)	σ_max_ (N mm^–2^)	ε_break_ (%)	*W* (N mm^–2^)	SRS (N mm^–1^)
ICol (non-cross-linked)	95.6 ± 0.8	5.0 ± 1.4	0.33 ± 0.06	17.1 ± 2.1	2.7 ± 0.4	0.3 ± 0.1
ICol (EDC/NHS cross-linked)	96.2 ± 0.7	11.7 ± 8.7	0.49 ± 0.30	16.8 ± 5.8	4.1 ± 2.3	0.4 ± 0.1
ICol-MA network (in acetic acid)	98.7^a^	7.6 ± 2.0	0.76 ± 0.34	52.7 ± 8.6	20.7 ± 12.2	0.3 ± 0.2
ICol-MA network (in DMSO/HCl)	96.2 ± 0.4	18.0 ± 6.8	0.75 ± 0.13	10.0 ± 3.9	3.3 ± 0.9	0.6 ± 0.1

a *n* = 1.

**Table 5:** Water Content and Mechanical Properties of Wet Non-Cross-Linked ICol and Networks Prepared from ICol and ICol-Ma

		**Tensile Properties**				
Material	Water Content(%)	*E*-modulus (MPa)	σ_max_ (N mm^–2^)	ε_break_ (%)	*W* (N mm^–2^)	SRS (N mm^–1^)
ICol (non-cross-linked)	4036 ± 281	0.05 ± 0.02	0.01 ± 0.003	51.1 ± 11.4	0.2 ± 0.1	0.01 ± 0.006
ICol (EDC/NHS cross-linked)	1902 ± 80	0.19 ± 0.05	0.03 ± 0.014	42.5 ± 7.9	0.7 ± 0.4	0.04 ± 0.02
ICol-MA network (in acetic acid)	2878 ± 164	0.91 ± 0.29	0.25 ± 0.12	74.1 ± 8.4	7.6 ± 3.2	0.2 ± 0.02
ICol-MA network (in DMSO/HCl)	2018 ± 341	0.27 ± 0.09	0.06 ± 0.02	62.5 ± 8.3	1.6 ± 0.6	0.2 ± 0.08

#### 3.5.2 Mechanical Properties

Table 4 provides an overview of the mechanical properties of the dry cross-linked ICol and ICol-MA networks, as well as that of the non-cross-linked ICol structure. Cross-linking improved the mechanical properties. The values of the modulus, maximum stress, toughness, and SRS of the cross-linked networks are all higher compared to that of the non-cross-linked structure. Curiously, the toughness of the ICol-MA network cross-linked in acetic acid solution is 8 times higher than that of the non-cross-linked structure and a 5 to 6 times higher compared to the other cross-linked networks.

The mechanical properties of the wet materials are listed in Table 5. In the wet state, the benefits of photo-cross-linking over the non-cross-linked ICol structure and the EDC/NHS-cross-linked ICol network become clear as well, in particular with respect to the increase in toughness and SRS. As the non-cross-linked ICol structure and the EDC/NHS-cross-linked ICol network are (initially) prepared in aqueous acetic acid, the contrast with the ICol-MA network prepared in the same solution shows the effect of photo-cross-linking itself. The tensile properties of this ICol-MA network are considerably improved compared to the other networks.

#### 3.5.3 Effect of the Solvent on Collagen

The considerable improvement of the mechanical properties of the ICol-MA network cross-linked in acetic acid solution was highly remarkable. This prompted us to investigate the effect of the solvent on the structure of the collagen.

Collagen fibrils have a distinct structural buildup. Three precursor polypeptide chains are twisted into a triple helix.^23^ Once the nonhelical telopeptides have been enzymatically cleaved, collagen fibrils are formed through self-assembly. The fibrils have a degree of regularity with a repeating periodicity of 67 nm. This periodicity, the D period, consists of a gap and an overlap region. It creates a distinct banding pattern that can be visualized not only by AFM but also by SEM; see Figure 3. We assessed the D-periodicity of ICol-MA swollen in acetic acid solution as part of the methacrylation, ICol-MA swollen in DMSO, and ICol-MA swollen in DMSO/HCl.

**Figure 3:**
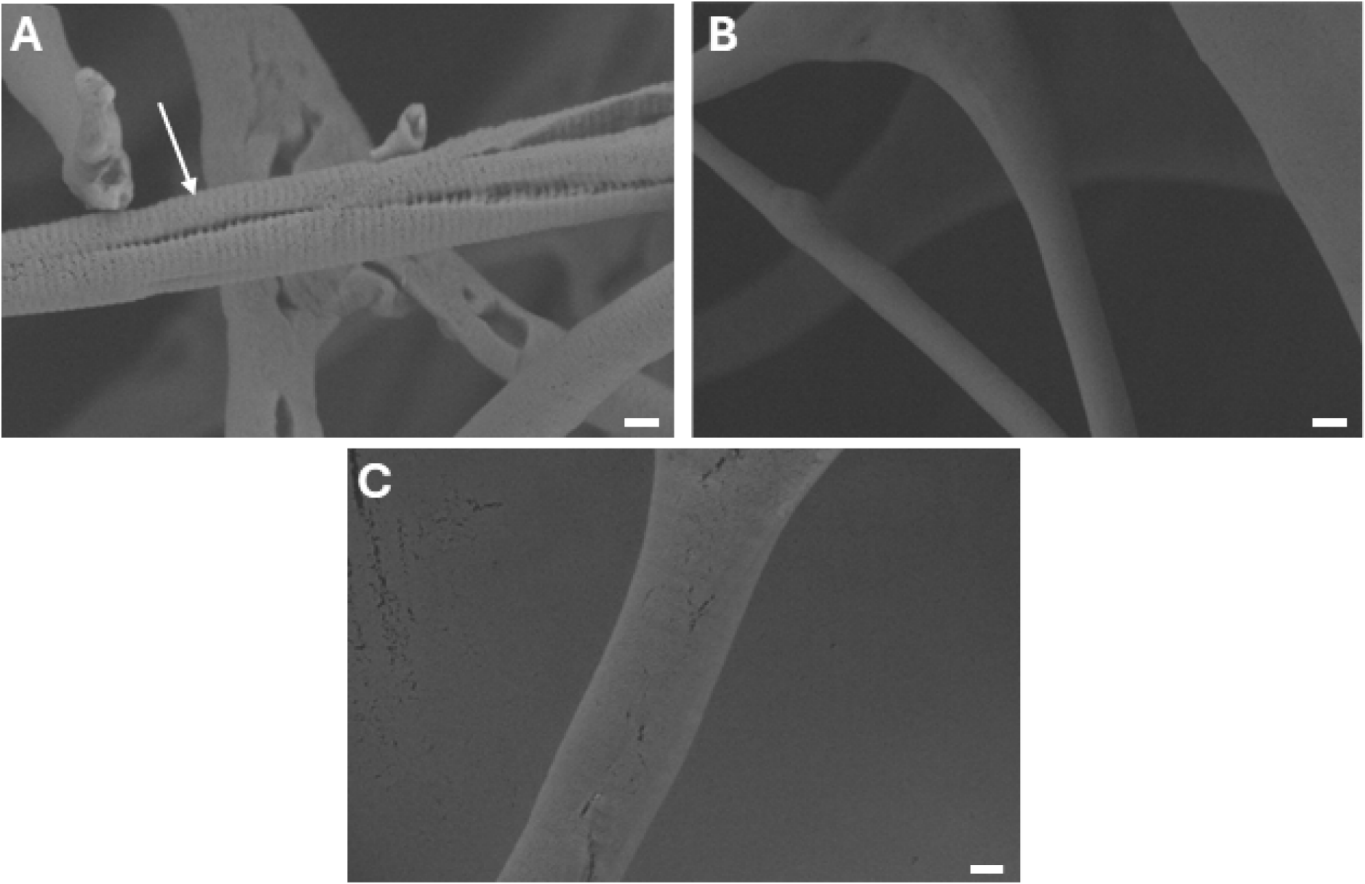
A) ICol-MA after swelling in acetic acid solution as part of the methacrylation reaction. The banding pattern is clearly visible; see the white arrow. B) ICol-MA after swelling in DMSO and subsequent washing and drying. The banding pattern is lost. C) ICol-MA after swelling in DMSO/HCl and subsequent washing and drying. No banding pattern can be observed. Scale bars are 200 nm.

Methacrylation of ICol does not affect this pattern, as can be seen in Figure 3A, indicating that the structure of ICol-MA is similar to that of ICol. During methacrylation, the collagen is swollen in acetic acid, indicating that this solvent does not affect the structure. After swelling in DMSO, the banding pattern is lost, as can be observed in Figure 3B. DMSO is an aprotic solvent with a high affinity for water and can penetrate the collagen, disturbing hydrogen bonds and water bridges responsible for its structure.^24^ That DMSO changes the structure of collagen has been shown;^25–27^ however, this change was reversible in some of these studies. Figure 3C shows that ICol-MA after swelling in DMSO/HCl does not have a banding pattern anymore. This shows that the nature of the solvent used has a large effect on the striation of collagen, which can be an important determinant in the mechanical properties of networks prepared in these solvents. To prepare hybrid networks, however, DMSO/HCl is essential as acetic acid solution is inappropriate to dissolve PTMC.

## 4 Conclusions

In this study, a method is presented to prepare hybrid networks from ICol and PTMC, resulting in highly porous networks due to DMSO crystallization. The obtained (hybrid) networks had high gel contents and compositions similar to those of the starting mixtures. Additionally, a higher collagen content increased the water content of water-swollen networks, as expected. The porous hybrid networks showed improved mechanical properties compared to a porous ICol-MA network and a non-cross-linked porous ICol structure. In particular, the ICol-MA:PTMC-tMA 17:83 wt % network showed promise for tissue engineering applications. We anticipate that the optimization of the composition of the hybrid scaffolds will further improve their toughness and SRS.

Photo-cross-linking considerably improved the mechanical properties of wet collagen networks, which is particularly interesting due to their relevance to the *in vivo* situation. ICol-MA networks photo-cross-linked in DMSO/HCl were compared to a more conventional EDC/NHS-cross-linked ICol network and an ICol-MA network photo-cross-linked in acetic acid solution. Interestingly, the toughness of the ICol-MA network photo-cross-linked in acetic acid solution was higher than that of the other collagen networks, indicating a potential effect of the solvent on the resulting mechanical properties. It was shown that acetic acid solution did not affect the collagen banding structure, while DMSO/HCl did. Further research into the effect of the solvent on network mechanical properties is required even though DMSO/HCl is essential for preparing hybrid networks.

## Supplementary Material



## Notes

Fe­(III)-assay calibration curve and TGA curves (PDF)
