# Supplementary material for: Photo-Cross-Linked Porous Hybrid Networks Based on Insoluble Collagen and Poly(trimethylene carbonate)
Source: Biomacromolecules. 2025 Jul 29;26(8):5399–408. doi: 10.1021/acs.biomac.5c00971 (PMC12344706; doi:10.1021/acs.biomac.5c00971)
Supplement: Supplementary file 1 [file bm5c00971_si_001.pdf]

Photo-crosslinked Porous Hybrid Networks based on Insoluble Collagen and Poly(trimethylene carbonate)

Bas van Bochove[1*], Lieke H.A. van Dommelen[2], Anne-Constance Macarez[1], Marc Ankoné[1], Elly M.M. Versteeg[2], Toin H. van Kuppevelt[2], Willeke F. Daamen[2], André A. Poot[1], Dirk W. Grijpma [1]

[1]Advanced Organ bioengineering and Therapeutics, Department of Bioengineering Technologies, Techmed Centre, University of Twente, Enschede, The Netherlands
[2]Department of Medical BioSciences, Research Institute for Medical Innovation, Radboud university medical center, Nijmegen, The Netherlands

Supplementary data

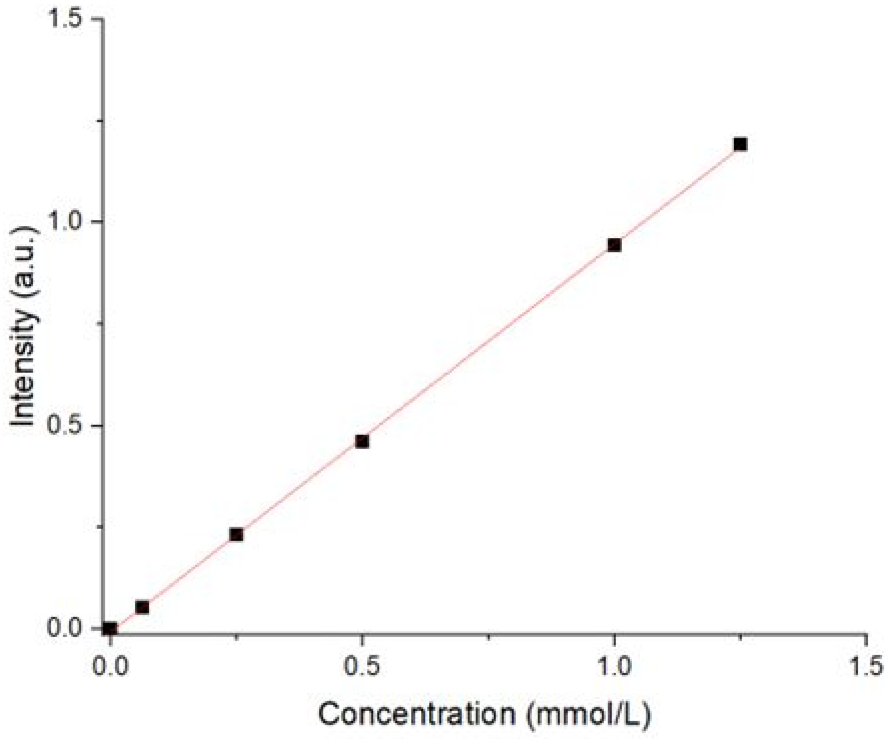

Figure S1. Calibration curve of the Fe(III)-assay to determine the degree of functionalisation of ICol-MA. Values are averages of three measurements. A linear fit (y=0,9541x -0,0073, in red) was found with an R2 value of 0,9998. Y-axis is the intensity of the absorbance at 500 nm and the x-axis represents the concentration of acetohydroxamic acid.

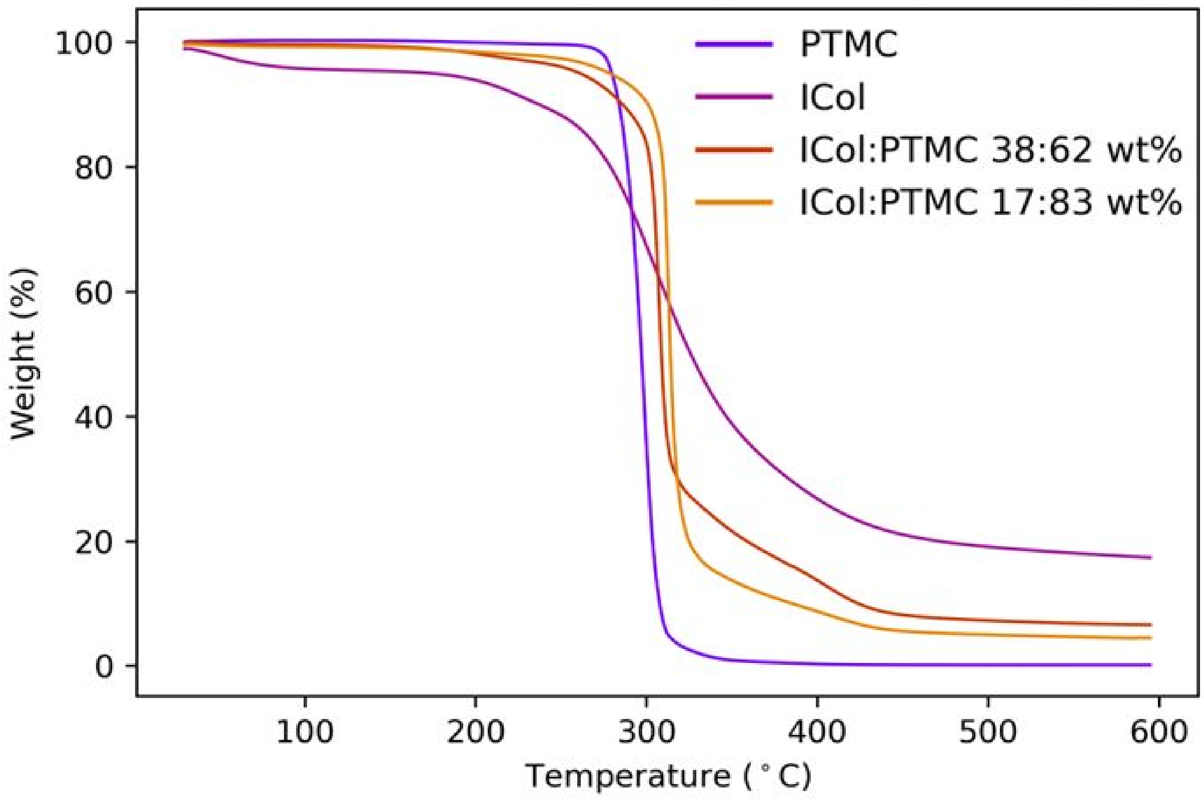

Figure S2. TGA-curves of the (hybrid) networks photo-crosslinked in DMSO/HCl.
